# An Embodied Paper‐Based Microfluidic Al‐Air Battery for Enduring Untethered Insect‐Scale Robots

**DOI:** 10.1002/advs.75702

**Published:** 2026-05-30

**Authors:** Yun Yang, Tao Jiang, Zhongyue Lu, Dapeng Fan, Zucheng Yi, Zirong Luo

**Affiliations:** ^1^ College of Intelligence Science and Technology National University of Defense Technology Changsha Hunan China; ^2^ National Key Laboratory of Equipment State Sensing and Smart Support National University of Defense Technology Changsha Hunan China; ^3^ Hunan Seirios New Material Technology Co.Ltd Changsha Hunan China

## Abstract

The scaling effects make energy supply a fundamental challenge for achieving autonomy in insect‐scale robotics. Inspired by the multifunctionality of biological tissue, dual‐function structural‐electrochemical integration based on a microfluidic aluminum‐air battery (MFAAB) paves a promising path toward energy autonomy in insect‐scale robotics. However, suboptimal mono‐surface anode utilization and restricted microfluidic transport dynamics of conventional AABs accelerate passivation and byproduct accumulation, hindering output performance. Here, we proposed and developed a centimeter‐size MFAAB with dual reaction surfaces for anode, and decompose byproduct accumulation via F^−^. Owing to the novel structural configuration and electrolyte optimization, it achieves a high capacity of 2697.05 mAh/g_Al_. The developed MFAABs demonstrate operational capacities of tens of milliwatts, successfully powering LEDs, DC motors, and electric toy car. We demonstrate the first successful integration of Al‐air batteries with insect‐scale robots through a structural‐electrochemical co‐design framework, harnessing energy weight proportion of 51.38%, achieving dual energy‐storage and electromechanical actuation functions for self‐sustaining operation. The integrated system simultaneously serves as a body‐conforming structural power source while exhibiting 2.56‐fold and 1.85‐fold higher endurance than a commercial lithium‐polymer (401015) and two series‐connected alkaline button batteries (LR44), respectively.

## Graphical abstract

This work presents a centimeter‐scale microfluidic aluminum–air battery (MFAAB) with dual‐surface anode and F^−^‐mediated byproduct decomposition. The design enables a high capacity of 2697.05 mAh/g Al and delivers tens of milliwatts to power LEDs, motors, and a toy car. Through a structural–electrochemical co design, the battery is integrated into an insect scale robot as a body conforming structural power source, achieving an energy weight proportion of 51.38%. The integrated system provides dual energy storage and electromechanical actuation, exhibiting 2.56 fold and 1.85 fold endurance improvements over a commercial Li polymer battery and two series connected alkaline button cells, respectively.

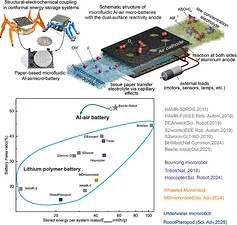

## 1 Introduction

The unique combination of multimodal locomotion [1, 2] and superior maneuverability [3, 4] in insect‐scale robots unlocks new possibilities for missions in confined and structurally sensitive environments. Unlike bulky machinery, these robotics can navigate complex terrains at high speeds with tight turning circles, making them ideal for demanding applications from precision inspection and exploration [5, 6, 7] to critical rescue operations [8, 9] and biomedical tasks [10, 11, 12]. The energy supply issue arising from scaling effects has become a critical bottleneck limiting insect‐scale robotics autonomous operation. Current primary energy solutions can be categorized into three types: (1) tethered power delivery via direct electrical wiring [13, 14, 15]; (2) Wireless power transfer using external energy sources such as light [16], magnetic fields [17], or electromagnetic waves [18]. However, these two approaches exhibit poor adaptability in complex environments, restricting the robot's freedom of movement [19, 20, 21]; And (3) involving onboard energy sources, such as lithium polymer batteries [4] or high‐energy chemical fuels [22], offering the highest potential for autonomy.

Owing to high theoretical specific energy and enhanced safety, metal‐air batteries such as aluminum, zinc, and magnesium, emerge as promising next‐generation energy storage system [23]. The unique open cathode architecture enables continuous oxygen supply from ambient air through gas diffusion electrodes, effectively eliminating the need for pre‐stored oxidants while maintaining compact system configuration [24, 25, 26, 27]. Naturally, their high theoretical energy density stems from the harmonious integration of the lightweight oxygen electrode and the high‐energy‐density metal anodes [28, 29, 30]. Among them, the aluminum‐air battery (AAB) stands out due to its high energy density (8.1 Wh/g) [31, 32], abundant reserves (constituting 8.2% of the Earth's crust), and sustainability. On the other hand, recycling societal waste aluminum can alleviate resource shortages and environmental issues by repurposing it into anodes [33]. Adipose tissue, ubiquitous in the bodies of organisms, has several functionalities such as energy‐storage, load bearing, and biochemical sensing [34]. Drawing inspiration from AAB and biological tissue, the substitution of conventional standalone batteries in robotic apparatus with structural‐electrochemical coupling in conformal energy storage systems can enhance insect‐scale robots’ performance through the simultaneous reduction in overall weight and extension of their power endurance [35].

However, a series of challenges bottlenecked the miniaturization and commercialization of AAB, such as immense liquid storage and transport systems for aqueous batteries [36], accumulation of the non‐conductive passivation layer for hydrogel‐based battery [37, 38], and severe Al self‐corrosion phenomenon [39]. The failure to swiftly remove insoluble byproducts on the anode's surface ultimately causing a blockage of the active sites. Cellulose paper has demonstrated tremendous potential in microfluidic batteries due to its unique capillary effect, particularly in replacing traditional pumping systems [31, 40]. The paper‐based AABs harness the natural water‐absorbing and liquid‐conducting capabilities of cellulose paper to achieve automatic circulation of electrolytes, thereby simplifying battery structures and reducing costs [41]. While pump‐free electrolyte flow has been achieved in certain microfluidic AABs, the constrained electrolyte transport capacity within microchannels induces anode passivation. Moreover, prevailing sandwich‐structured MFAABs—composed of sequentially stacked aluminum anode, microchannel separator, and air cathode exhibit inefficient material utilization, rapid capacity fading, and performance degradation due to the single reactive surface configuration of the batteries [42, 43, 44].

A few studies have explored dual‐reaction‐surface anode designs to enhance the utilization efficiency of aluminum anodes by increasing the electrochemically active area [45]. For instance, Liu et al. [46] demonstrated that a double‐sided anode configuration in microfluidic batteries could effectively improve reaction kinetics and delay passivation. On the other hand, the accumulation of insulating byproducts (Al(OH)_3_) on the anode surface remains a fundamental challenge that limits long‐term operation. To address this issue at the material level without compromising the system's structural simplicity, Wei et al. [47]. introduce a chemical approach by employing fluoride ions (F^−^) in the electrolyte. F^−^, possessing a similar ionic structure to OH^−^ but with a smaller ionic radius and higher charge density, can competitively adsorb onto the aluminum surface. This interaction is expected to suppress the formation and accumulation of the passivating Al(OH)_3_ layer, thereby maintaining active site accessibility.

**FIGURE 1:**
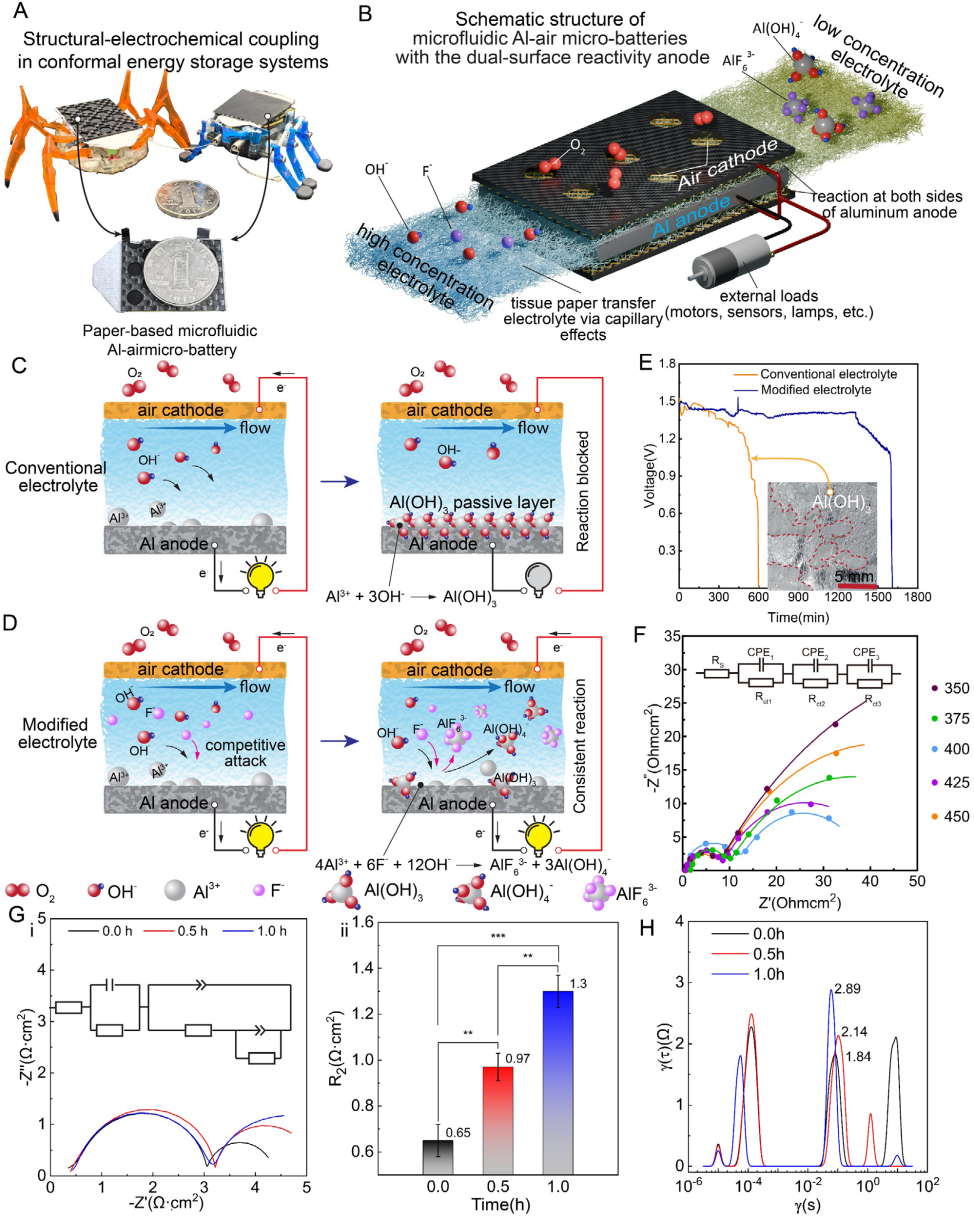
Structural‐electrochemical coupling in conformal energy storage systems. (A) Structural‐electrochemical coupling in conformal energy storage systems for miniaturized biomorphic robots. (B) Schematic structure of microfluidic Al‐air micro‐batteries. (C)Anode passivation mechanism in conventional aluminum‐air batteries. (D) Passivation layer formation suppression mechanism via modified electrolyte. (E) Constant‐current discharge curve at 10 mA/cm^2^ for batteries with modified and conventional electrolyte. Inset image demonstrates the insoluble reaction products on the anode for the conventional electrolyte system. (F) EIS fitting curve and equivalent circuit. (G) EIS anode with different time. (H) Distribution of Relaxation Times (DRT).

Focus on the deep integration of onboard energy systems with the robotic structure, we developed a structural‐electrochemical coupling mechanism based on MFAAB for insect‐scale robots (Figure 1A). This system not only provides energy storage but also possesses mechanical load‐bearing capacity, thereby achieving functional integration at both the energy and structural levels. In this work, we report a centimeter‐size MFAAB featuring a dual‐reaction‐surface anode—a design that has been previously explored in conventional microfluidic configurations but is here adapted and optimized for paper‐based systems to improve anodic utilization and power output. Furthermore, we introduce F^−^ into the electrolyte to suppress parasitic byproduct accumulation, building upon prior findings while demonstrating its enhanced efficacy in the confined microchannel environment of paper‐based batteries (Figure 1B). The battery performance is systematically optimized by investigating the effects of different Al anode materials, separator materials, inter‐electrode distance, and electrolyte concentrations. The results show that this MFAAB exhibits a high peak power density of 45.73 mW/cm^2^, an energy density of 3371.37 mWh/g_‐Al_ and enable to power LEDs, DC motors, and mini‐EVs. Furthermore, inspired by insect morphology, we demonstrate an unprecedented body‐integrated structural MFAAB for insect‐scale robot. This bioinspired design achieves a 12.48‐fold and 3.18‐fold volumetric capacity enhancement over conventional standalone lithium‐polymer and alkaline button batteries of equivalent size, highlighting the novelty and significance of our structural‐electrochemical integration approach.

## 2 Results

### 2.1 Parasitic Reaction Suppression Mechanism

In the context of aqueous aluminum‐air batteries, a peristaltic pump serves as a crucial component for inducing the circulation of the electrolyte within the battery system. This cyclic flow mechanism effectively promotes the detachment of reactive byproducts (especially Al(OH)_3_) from the electrode surfaces, ensuring the cleanliness and activity of the electrode interface. In our design, capitalizing on the capillary effect, we have achieved the circulation of the electrolyte without the necessity of a peristaltic pump as a driving force. Despite this approach is innovative, the limited capacity of the separator to transport the electrolyte poses a significant challenge. Consequently, insoluble discharge products cannot be efficiently removed, clogging anode‐electrolyte ion channels and thereby delaying anodic oxidation while shortening discharge duration, showing in Figure 1C. Owing to comparable structural compatibility, reduced ionic radius, and enhanced charge density, F^−^ acts as a competing species against O^2^^−^. and exhibits a greater tendency to form stable complexes or ion pairs with cations or solvent molecules in chemical reactions. The electrostatic cohesion between O^2−^ anions and Al^3+^ cations within the crystalline lattice of Al(OH)_3_, comprising the passivation layer, is attenuated upon the adsorption of F^−^ ions onto its superficial domain. This weakening of electrostatic forces facilitates the preferential coordination of F^−^ ions with Al^3+^ ions, leading to the synthesis of the K_3_AlF_6_ complex (Equation (1)), which exhibits exceptional thermodynamic stability and heightened solubility [47]. Figure 1D elucidates how F^−^ ions enhance battery durability through competitive adsorption at Al anode interfaces: By preferentially occupying surface sites, they simultaneously block Al(OH)_3_ nucleation pathways and delay passivation dynamics.

(1) $$\begin{array}{ccc} & & 4Al^{3+}+6F^{-}+12OH^{-}\to Al{F_{6}}^{3-}+3Al{{\left(OH\right)}_{4}}^{-}\;\;\\ & & \Delta_{r}G^{\circ }=-435.72kJ\cdot mol^{-1}<0 \end{array}$$

Constant‐current discharge tests performed at 10 mA/cm^2^ for both battery configurations (Figure 1E) demonstrated that the modified electrolyte‐based AAB achieved a discharge duration of 1600 min, corresponding to 2.70‐fold prolongation compared to the conventional electrolyte system. Notably, extensive insoluble reaction products were detected on the anode surface of conventional electrolyte batteries during operation (Figure 1E in‐line optical photograph). Complementary linear voltammetry analyses (Figure S1A) further corroborated this trend, revealing a peak power density of 45.67 mW/cm^2^ for the modified MFAAB — a 1.5‐fold enhancement relative to the conventional M‐FAAB. These findings collectively demonstrate that F incorporation not only extends cycling stability but also significantly improves electrochemical activation kinetics.

Furthermore, the EIS of the battery is conducted to investigate electron transfer and phase diffusion on the anode surface. Figure 1F exhibits the Nyquist diagram of D‐MFAAB with various electrolyte concentrations at the stable OCV, which is composed of four parts. The first is R_s_, with little difference for D‐MFAAB with different electrolyte concentrations (Table S1). The second part is the high‐frequency capacitive‐reactance arc, which represents the initial formation of the passivation layer on the Al surface. The third comprises the intermediate‐frequency capacitive‐reactance and inductive‐reactance arcs, which are crucial steps in anodic oxidation, representing the process of Al↔Al^+^ and the adsorption/desorption of parasitic product Al(OH)_3_ respectively. Both R_ct1_ and R_ct2_ follow a similar trend where they decrease gradually as the concentration of the electrolyte increases, but exhibit a slight increase beyond 400 g/L (Table S1). The results indicate that the introduction of fluorine disrupts the formation of the passivation layer, thereby facilitating ion transport channels and mitigating the inhibitory effect of Al(OH)_3_ on anodic oxidation [47]. The fourth is the low‐frequency capacitive‐reactance arc, which represents the continuous oxidation of Al+↔Al^3+^. The R_ct3_ for each no longer has a significant difference. Finally, an equivalent circuit that can reflect the regularity of interfacial electron transfer is shown in Figure 1F.

Furthermore, comparative analysis of the anode's Nyquist plots at varying test durations (Figure 1G i) consistently revealed distinct “two short semicircles” features. Based on the measured frequency ranges and variations in radii, interpretation confirms that the two semicircles correspond to distinct processes: the high‐frequency short semicircle represents the double‐layer capacitance at the electrolyte/aluminum anode interface and the charge transfer process of Al → Al^3^^+^. Its measured radius remained stable within 1.29–1.33 Ω, showing no significant variation with testing time, with a maximum fluctuation of ≤0.04 Ω (Figure S1B), indicating that the charge transfer rate and associated resistance remained stable without notable fluctuation. The mid‐frequency short semicircle is associated with the Al ↔ Al^+^ redox reaction and the adsorption/desorption processes of Al(OH)_3_ on the aluminum anode surface. As the testing time extended, the radius of this semicircle increased steadily from 0.65 to 1.3 Ω, with a cumulative increase of 0.65 Ω. The increments were uniform across time intervals, consistent with the increasing adsorption of Al(OH)_3_ and the progressive accumulation of Al ↔ Al^3+^ reaction products (Figure 1G ii). Complementary evidence from DRT (Distribution of Relaxation Times) analysis (Figure 1H) showed that the relaxation peak corresponding to the mid‐frequency region (τ = 10^−2^–10^0^ s) intensified significantly with prolonged testing time, corroborating the observed changes in the EIS spectra.

### 2.2 Design Strategy and Assembly Process for Paper‐Based Microfluidic Al‐Air Micro‐Batteries

As shown in Figure 1B, we have designed an aluminum‐air battery schematic featuring intrinsic electrolyte migration, which comprises a multi‐functional air cathode, a porous cellulose separator, and an Al anode, stacked horizontally in a sandwich configuration from top to bottom. The inlet of the separator is securely anchored within the electrolyte storage tank, ensuring its complete immersion in the electrolyte. Furthermore, the outlet is positioned strategically at the apex of the effluent tank, ensuring that there is no direct interaction between the separator and the waste liquid, thereby maintaining the integrity and functionality of the separator. Leveraging the inherent capillary action of the separator, the electrolyte was autonomously transported from the anodic chamber through the separator interface, subsequently diffusing through the porous architecture of the aluminum‐air cell via wicking mechanisms, and ultimately accumulating in the designated effluent reservoir. Concurrently, the separator performs the crucial function of isolating the anode and cathode, thus mitigating the risk of short circuits within the battery, and ensuring its safe and efficient operation.

**FIGURE 2:**
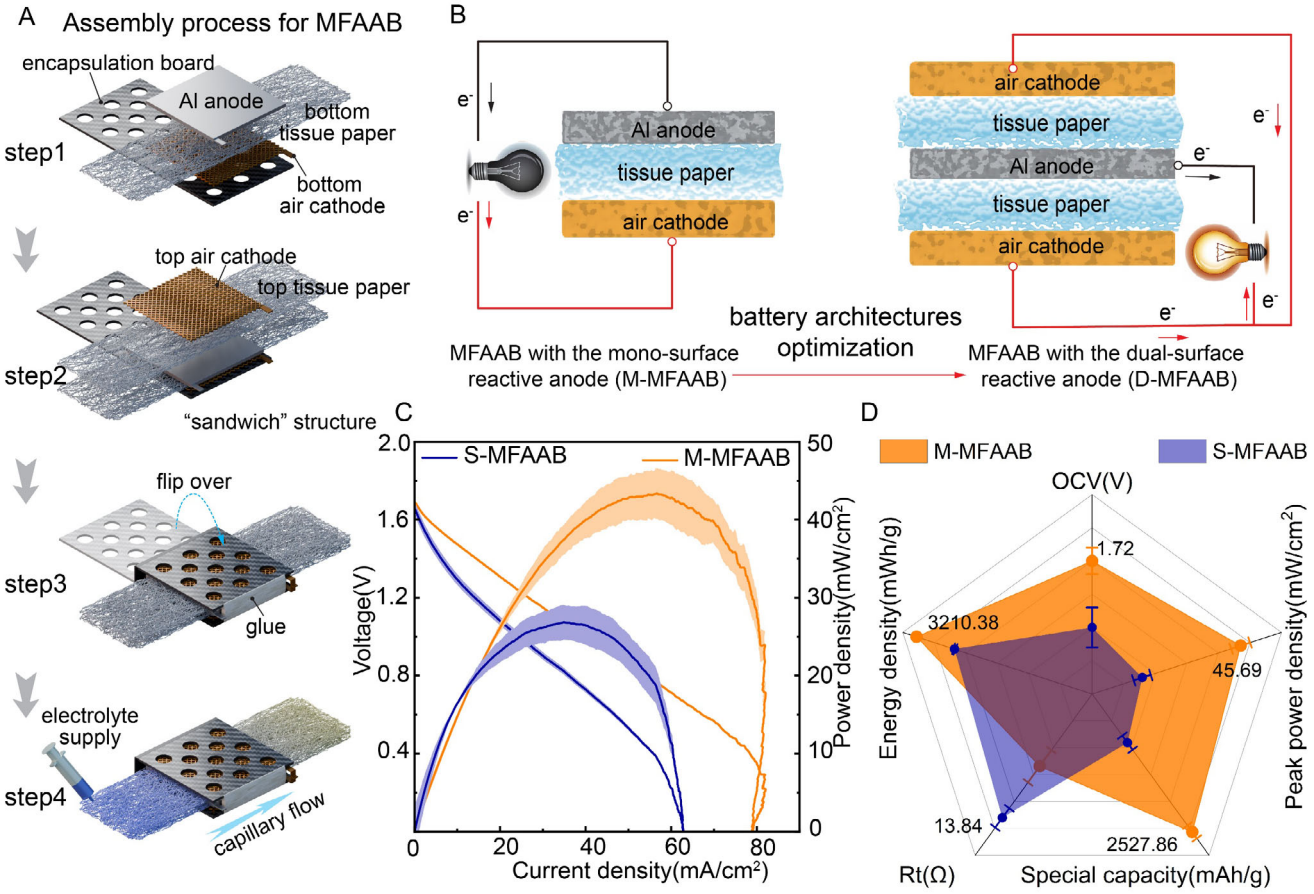
Design concepts and performance comparison for MFAAB. (A) Assembly process schematic diagram. (B) Internal architectural details for M‐MFAAB and D‐MFAAB. (C) Polarization curves and density curves (data are presented as mean ± SD with n = 3). (D) Key performance comparison.

Having leveraged the energy conversion mechanism, we have devised two Al‐air batteries: the one with the mono‐surface reactive anode (M‐MFAAB), and the other with the dual‐surface reactive anode (D‐MFAAB), visually presented in Figure 2B. The M‐MFAAB was composed of an upper cover plate with holes, an air cathode, a porous paper‐based separator, an Al anode, and a bottom plate. The AAB employs a polyimide (PI) flexible hinge mechanism coupled with carbon fiber‐reinforced panels to realize three‐dimensional packaging. Specifically, a PI film pivot structure is engineered at the terminal of the packaging unit's symmetric axis. During assembly, the lower substrate remains stationary while the upper clamshell undergoes 180° rotation about the hinge axis, enabling face‐to‐face alignment of the upper and lower clamshells to achieve structural consolidation. At the opposing symmetric axis terminus, epoxy resin adhesive bonding is implemented between the clamshells, culminating in an integrated battery configuration. In conclusion, the detailed manufacturing process of the equipment is illustrated in Figure 2A. Distinct from the M‐MFAAB's unipolar configuration, the D‐MFAAB achieves a bipolar configuration through sequential integration of a composite separator and gas diffusion cathode onto the anode. This engineered design establishes dual electrochemically active interfaces, enabling simultaneous metal oxidation reactions on both surfaces of the anode within a unified cell architecture.

The performance comparison between the M‐MFAAB and D‐MFAAB is shown in Figure 2C. The *I*–*V* curve exhibits a classic linear decrease, while the power density curve (I‐P) follows a characteristic parabolic shape, which exactly corresponds to the relationship *P* = *U* × *I* = *I* ^2^ × *R* = *E* × *I* − *I* ^2^ × *r* (where *U* = *E* − *I* × *r*). At the end of the scan, the *I*–*V* curve deviates from linearity to distorted or sharply bent tails due to the depletion of hydroxide ions near the electrode interface in the high‐current‐density region, as confirmed by the single‐electrode scan in Figure S1C. For the experiment, we employed the Al_3N_ anode, GCP separators, and the 400 g/L electrolyte. As shown in Figure 2C, the performance of D‐MFAAB is superior to that of the M‐MFAAB: the peak power density of the D‐MFAAB is 45.69 mW/cm^2^, which is about 1.29 times that of the M‐MFAAB (35.33 mW/cm^2^). The maximum current density of the D‐MFAAB is 86.73 mA/cm^2^, which is 1.06 times that (81.92 mA/cm^2^) of the M‐MFAAB. A pronounced current reverse is observed in the D‐MFAAB at low operating voltages (Figure 2C), signaling the onset of severe mass transport limitations at the anode. This behavior is consistent with the single‐electrode polarization characteristics shown in Figure 1C. While the air cathode maintains stable linear polarization across the tested range—reflecting unimpeded oxygen reduction kinetics—the aluminum anode exhibits marked polarization at elevated current densities, characteristic of diffusion‐limited operation. The underlying cause is the restricted OH^−^ supply within the microfluidic architecture: the finite electrolyte volume limits the total OH^−^ reservoir, while the confined microchannel geometry hinders diffusive transport to the anode surface. At high current densities, the rate of OH^−^ consumption exceeds its diffusive replenishment, leading to localized OH^−^ depletion, increased concentration overpotential, and ultimately the observed current reverse.

The EIS measurements for the M‐MFAAB and D‐MFAAB at the OCV are carried out to adequately explain the electricity generation performance difference. And the test results and the fitting curves of the equivalent circuit are shown in Figure S2A, the R_s_ of the D‐MFAAB is 0.40 Ω cm^2^, lower than that of the M‐MFAAB (1.13 Ω cm^2^). In the Nyquist plot of the high‐frequency region, the semicircle diameter of M‐MFAAB is conspicuously larger than that of D‐MFAAB, clearly demonstrating the R_ct_ (10.65 Ω cm^2^) is more than that of the D‐MFAAB (5.53 Ω cm^2^). This elevated R_ct_ signifies an increased impediment to the flow of charge carriers during electrochemical processes within the battery, which can potentially diminish its power generation performance and overall efficiency. The primary reason for this phenomenon lies in the increased electrode reaction surface area, which significantly enhances the electrode reaction kinetics.

We selected the linear portion of the U‐I curves from Figure S2B and utilized Equation (2) to individually calculate the Rt for M‐MFAAB and D‐MFAAB. Although the Rt of the D‐MFAAB (13.84 Ω m^2^) is slightly larger than that of M‐MFAAB (12.24 Ω m^2^), D‐MFAAB exhibits a significant higher activation level compared to M‐MFAAB. Figure S2C shows the discharge curves of M‐MFAAB and D‐MFAAB at a current density of 20 mA/cm^2^. The average discharge voltage (1.08 V) and operation time (11.75 h) of the DR‐Al‐air battery are higher than those of M‐MFAAB (0.97 V, 9.72 h). According to Equations (3)–(5), we have calculated three pivotal performance metrics for the two batteries: specific capacity, anode efficiency, and energy density. After calculation, we have determined that M‐MFAAB and D‐MFAAB exhibit specific capacities of 2251.17 and 2527.86 mAh/g_‐Al_, respectively. Additionally, their anode efficiencies are 75.54% and 84.83%, while their energy densities stand at 2183.63 and 2730.09 mWh/g_‐Al_, respectively. After discharging, much M‐MFAAB anode material remains unused, causing waste, as shown in Figure S2D. To sum up, although the structure of the D‐MFAAB is slightly more complex than that of the M‐MFAAB, its electric generation and discharge performance are significantly superior to that of the M‐MFAAB (Figure 2D), we've chosen the D‐MFAAB for in‐depth research.

### 2.3 Al Anode

The Al anode material is a key factor affecting the power production of AABs. Three aluminum anodes were fabricated through a series of processing steps, including doping, rolling, and annealing. The introduction of trace non‐metal elements (e.g., B, Si) served as heterogeneous nucleation sites, effectively suppressing excessive grain growth during solidification. This process refined the originally coarse equiaxed/columnar grains into a fine and uniform equiaxed microstructure. Concurrently, these dopants tended to segregate at grain boundaries, altering their chemical composition and structure—for instance, by reducing the segregation of detrimental impurities or forming stable secondary phases—thereby contributing to grain boundary strengthening.

Subsequent rolling induced plastic deformation, whereby extensive slip of atoms along crystallographic planes led to the proliferation and entanglement of dislocations. This resulted in a high‐density deformation structure, effectively fragmenting the original grains at the microscale. The ensuing annealing treatment provided the necessary thermal activation energy to relieve internal stresses and drive the recrystallization process. This process involves the formation and growth of new, strain‐free equiaxed grains from the deformed matrix, culminating in a significantly optimized microstructure.

**FIGURE 3:**
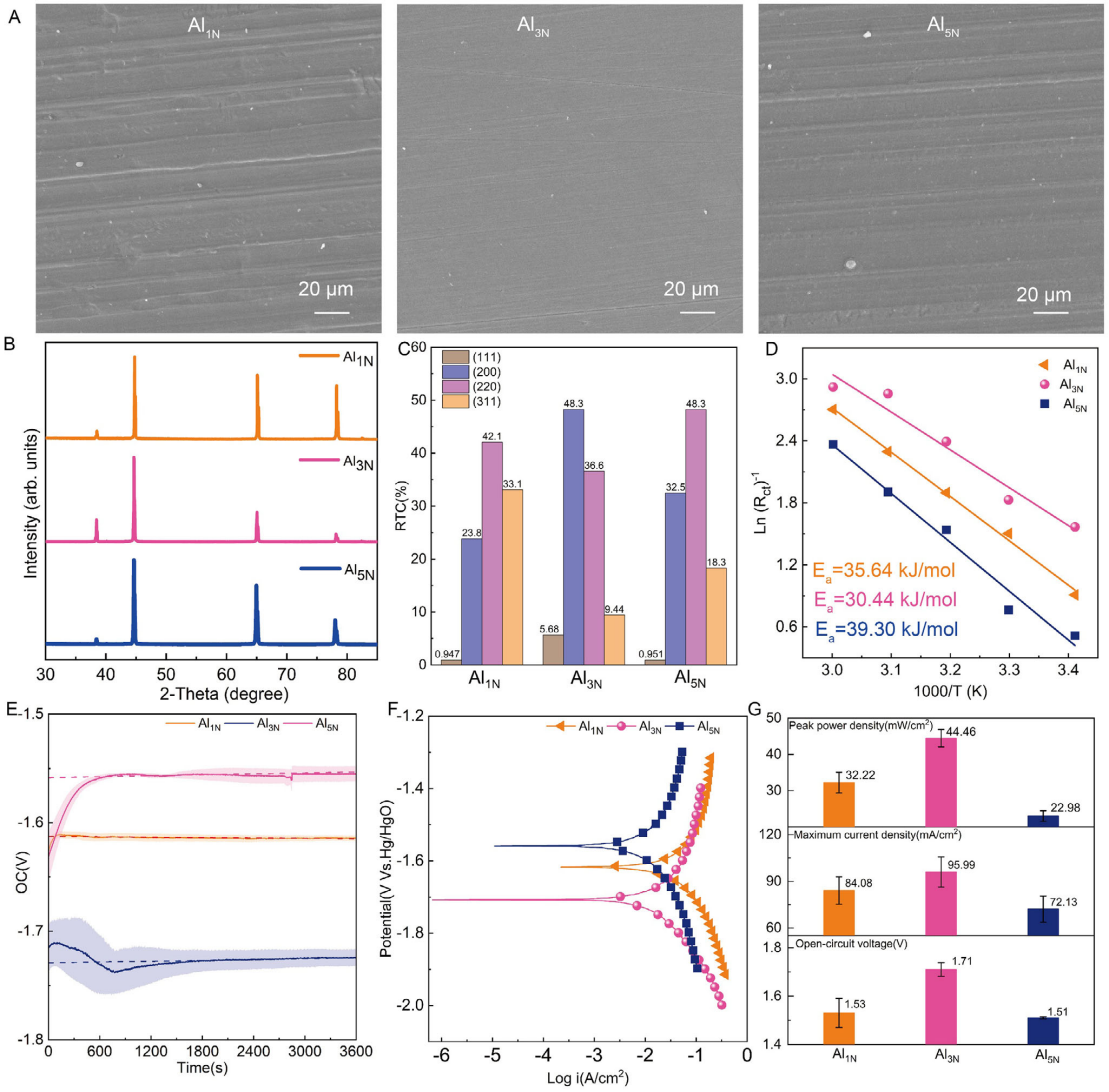
Selection principle for optimal Al anode. (A) SEM images, (B) XRD patterns, and (C) histograms for the corresponding fitted RTC. (D) The activation energies, (E) OCP and (F) potentiodynamic polarization curves of the different aluminum anodes. (G) Polarization curves of M‐MFAAB with the different aluminum anodes.

Figure 3A presents surface SEM images of the three aluminum foils with different preferred orientations, revealing slight variations in their surface texture. In addition to microstructural morphology, the crystal structures of the anodes were probed by x‐ray diffraction (XRD). As shown in Figure 3B, all three anodes exhibited distinct diffraction peaks at 2θ angles of approximately 38.4°, 44.7°, 65.1°, and 78.2°, which are indexed to the (111), (200), (220), and (311) planes of face‐centered cubic (FCC) aluminum, respectively. These peak positions are in good agreement with the standard reference data (PDF#85‐1327). Based on their purities, the anodes were designated as Al_1N_ (∼97% Al), Al_3__N_ (∼99.9% Al), and Al_5N_ (high‐purity grade, ∼99.999% Al).

To quantitatively evaluate the preferred crystallographic orientation, the relative texture coefficient (*RTC*) for each diffraction plane was calculated using the following formula:

(2) $$RTC_{\left(hkl\right)}=\frac{I_{\left(hkl\right)}/I_{0(hkl)}}{\sum I_{\left(hkl\right)}/I_{0(hkl)}}\times 100\%$$

where *I*_(*hkl*)_ and *I*_0(*hkl*)_ represent the measured and standard reference diffraction intensities for the (*hkl*) plane, respectively. The results further quantify the differences in preferred crystallographic orientation among the anodes, with (*RTC*) values of 42.1%, 48.3%, and 48.3% for the Al_1N_,Al_3N_, and Al_5N_ anodes, respectively (Figure 3C and Table S2).

We next examined the kinetics of the three aluminum anodes in a liquid electrolyte. Figure S3C presents electrochemical impedance spectra of symmetric cells assembled with Al anodes of different preferred orientations, measured at various temperatures following cyclic voltammetry tests (fitted data are summarized in Table S3). The Al_3N_ anode consistently exhibited the lowest charge‐transfer resistance across the tested temperatures, indicating a markedly enhanced electrochemical stripping rate compared to the other anodes.

The kinetics of the electrochemical stripping process is governed by its activation energy. The apparent activation energy (Ea), representing the energy barrier of the rate‐determining step for the stripping reaction, was determined for each textured anode from the temperature‐dependent data using the Arrhenius equation:

(3) $$\frac{1}{R_{ct}}=Ae^{-\frac{E_{a}}{RT}}$$

(4) $$\ln\left(R_{ct}^{-1}\right)=-\frac{E_{a}}{R}\times \frac{1}{T}+\ln A$$

(5) $$k=-\frac{E_{a}}{R}$$

(6) $$E_{a}=-kR$$

Here, *R_ct_* represents the charge transfer resistance, *A* is the pre‐exponential factor, *R* is the universal gas constant, and *T* is the absolute temperature. As shown in Figure 3D, the calculated activation energies for the Al_1N_ and Al_5N_ anodes were 35.64 and 39.30 kJ mol^−^^1^, respectively. In contrast, the Al_3N_ anode exhibited a lower activation energy of 30.44 kJ mol^−^^1^, indicating superior charge‐transfer kinetics.

The negative shift in the open‐circuit potential (OCP) serves as a critical indicator of enhanced electrochemical activity in anode materials. As depicted in Figure 3E, among aluminum anodes of varying purity levels, the Al_3N_ electrode exhibits the most negative OCP value of −1.73 V (vs. Hg/HgO), which is considerably lower than those of the Al_5N_ (−1.56 V) and Al_1N_ (−1.61 V) anodes. This trend suggests that the incorporation of specific trace elements effectively facilitates the depassivation of the anode surface, leading to increased susceptibility of the protective oxide layer to fracture. As a result, the active dissolution of the metal is promoted, shifting the mixed potential negatively. These findings are in strong agreement with the reduced activation energy barrier for anodic dissolution derived from electrochemical kinetic analysis in Figure 3D, collectively affirming the role of trace elements in optimizing the electrochemical activity of aluminum anodes.

Analysis of the Potentiodynamic Polarization Curves (Figure 3F) and the Corresponding Parameters (Table 1) reveals a distinct electrochemical signature for the high‐purity aluminum anodes (Al_3N_ and Al_5N_). A significantly enlarged anodic Tafel slope (βa) points to the effective suppression of anodic dissolution, a consequence of the formation of a more passivating surface film. Conversely, the reduced cathodic Tafel slope (|βc|) suggests facilitated kinetics for the hydrogen evolution reaction (HER). Critically, the inhibitory effect on the anodic reaction is the dominant factor governing the overall corrosion behavior. This is unequivocally demonstrated by the corrosion current density (Icorr), derived via Tafel extrapolation, which is substantially lower for both Al_3N_ and Al_5N_ compared to the unpurified (Al_1N_) benchmark. We therefore conclude that enhancing the purity of the Al anode effectively blocks the anodic dissolution pathway. The net result of this strategic inhibition is a markedly suppressed corrosion rate and a superior electron utilization efficiency, as the flow of electrons is diverted toward the external circuit rather than being consumed by parasitic hydrogen evolution.

**TABLE 1:** Open circuit potential and corrosion parameters for anode.

Anode	Ave. Eocp (vs.Hg/ HgO)/V	E_corr_ (vs. Hg/HgO)/V	I_corr_ (mA/cm^−2^)	R_p_(Ωcm^2^)	|β_c_| (mVDec^−1^)	β_a_(mVDec^−1^)
Al_1N_	−1.62	−1.62	14.13	2.61	196.77	166.73
Al_3N_	−1.71	−1.72	5.13	5.92	150.52	171.17
Al_5N_	−1.56	−1.56	3.72	9.92	181.27	204.19

In Figure 3G, the performance of batteries that use Al_3N_ anodes was considerably better than that of batteries that use Al_1N_ and Al_5N_: (1) Notably, the peak power density reaches 44.46 mW/cm^2^ for Al_3N_—1.50 times that of Al_1N_ (32.22 mW/cm^2^) and 2.05 times that of Al_5N_ (22.98 mW/cm^2^). (2) A broader current output range is also achieved with Al_3N_ (0.05–98.99 mA/cm^2^), surpassing that of both the Al_5N_ anode (0.01–84.08 mA/cm^2^) and the Al_1N_ anode (0.01–72.13 mA/cm^2^) (Figure S3A). (3) Complementing these advantages, the open‐circuit voltage (OCV) of the Al_3N_‐based battery reaches 1.71 V, markedly higher than the 1.53 V recorded for Al_1N_ and 1.51 V for Al_5N_.

Figure S3C shows the electrochemical impedance spectroscopy (EIS) results of the full battery assembled with Al_1N_, Al_3N_, and Al_5N_ electrodes as negative electrodes, respectively. The fitting results documented in Table S4 quantify the charge transfer resistance (R_ct_) of Al_3N_ at 35.27 Ω cm^2^, representing a 48.7% reduction compared to Al_1N_ (68.8 Ω cm^2^) and Al_5N_ (69.52 Ω cm^2^) counterparts. Comparative SEM analysis in Figure S3D–F reveals distinct morphological evolution of three anode materials subjected to 1‐hour discharge at 10 mA cm^−^^2^. The Al_3N_ surface exhibited substantial delamination debris, with crack widths (2.88 µm) significantly exceeding those observed in Al_1N_ (1.29 µm) and Al_5N_ (0.93 µm) variants. Those indicate that a full battery assembled with Al_3N_ electrodes exhibits faster Al stripping reaction kinetics [48]. Although Figure 3G and Figure S3B,C indicate that the Al_5N_ anode has smaller R_s_ (0.34 Ω cm^2^ ) and Rt (12.74 Ω cm^2^) compared to the Al_3N_ anode (0.38 Ω cm^2^, 14.00 Ω cm^2^) and Al_1N_ anode (0.61 Ω cm^2^, 14.36 Ω cm^2^), the trace elements (such as Si and P) contained in Al_3N_ anodes contribute to enhancing the anode activity, facilitating electron lose and electrochemical reactions of Al anode, ultimately generating higher output voltage and power. Lastly, we opted to the Al_3N_ anode for further research to ensure the battery achieves high performance.

### 2.4 Separator Materials and Inter‐Electrode Distance

**FIGURE 4:**
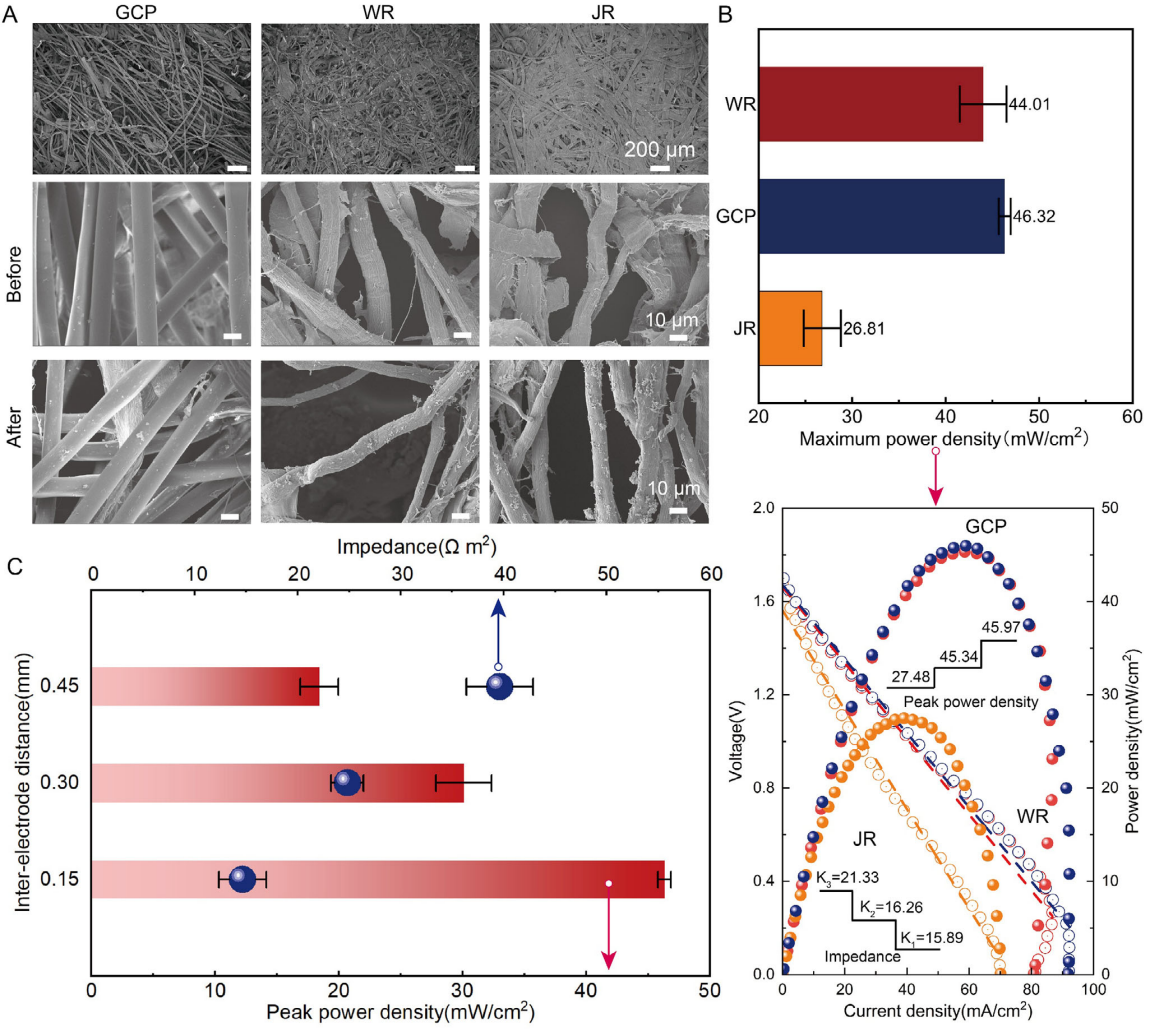
Performance of D‐MFAAB with different separators and inter‐electrode distance. (A) Separator SEM images before and after discharge. (B) Power density curves and polarization curves. (C) Peak power density and impedance for different inter‐electrode distance.

The separator materials serve as a pivotal factor that significantly influences the power generation efficiency in microfluidic Al‐air batteries. In contrast to traditional separator materials like polypropylene, polyvinylidene fluoride (PVDF) membranes, and ion‐exchange membranes, we opted for cost‐effective hydrophilic porous materials like kitchen and cleaning paper as battery separators. Upon assessing their power generation capabilities, we confirmed the viability of hydrophilic porous materials as separators for Al‐air batteries. In comparison to the haphazard arrangement of WR and JR fibers, GCP boasts uniformly sized and orderly arranged fibers (Figure 4A), which greatly facilitates the transport of electrolytes. Linear sweep voltammetry (LSV) testing results revealed that the GCP (46.32 mW/cm^2^) and WR (44.01 mW/cm^2^) electrode exhibited superior electrogenic performance compared to JR (26.81 mW/cm^2^), as evidenced by their enhanced peak power density and short‐circuit current density response, illustrated in Figure 4B. The EIS results (Figure S4A) and polarization curves (Figure 4B) showed that the GCP had the lowest R_s_ (0.45 cΩ m^2^) R_ct_ (5.58 cΩ m^2^) and R_t_ (14.00 Ω m^2^) compared to that of other separators (Table S5).

As shown in Figure S4B and Table S6, the electrolyte storage capacities are 9.24 for GCP, 9.07 for WR, and 7.94 for JR. This indicates that for the same weight of separator material, the GCP can store more electrolyte inside the pores of the material, facilitating the interfacial charge transfer between Al and electrolyte. Furthermore, the GCP separator performed better because of its highest thickness of 0.15 mm compared to 0.17 mm of JR and 0.16 mm of WR. The thin separator can minimize electrode distance, resulting in a substantial decrement of R_t_ (Figure 3E) and improved power production. In addition, Figure 4A compares the fiber changes of three separators before and after being immersed in electrolyte for 10 h. It reveals that the fibers of GCP remain relatively intact, while those of WR and JR exhibit varying degrees of damage and fracture. GCP maintains the integrity of its fibers when immersed in electrolyte and exhibits excellent wettability and strong capillarity. These properties significantly contribute to driving the pump‐free microflow within the separator material, facilitating the real‐time replenishment and renewal of electrolyte, and effectively enhancing the mass transfer process between the electrolyte and electrode.

The inter‐electrode distance manifests dual effects on battery performance through distinct mechanisms. (1) Reduced ionic transport resistance: Thinner inter‐electrode distance architectures minimize the electrolyte ionic transport pathway, effectively lowering mass transport resistance and enhancing interfacial charge transfer kinetics, as evidenced by electrochemical impedance spectroscopy (EIS) analysis (Figure S5A); (2) Enhanced electrolyte buffering capacity: Conversely, thicker inter‐electrode distance configurations retain greater electrolyte volume, maintaining sufficient hydroxide ion reservoirs during prolonged discharge cycles, thereby improving depth‐of‐discharge and cycle stability(Figure S5B).

As observed in Figure 4C, when inter‐electrode distance (IED) is 0.152 mm, the battery encounters a current reversal in the low‐voltage region, indicating insufficient hydroxide supply to the anode reaction surface. This issue is alleviated by adopting a 0.30 mm thick IED, yet it comes at the cost of a decrease in peak power density from 45.35 to 31.79 mW/cm^2^. An increment of the IED to 0.45 mm would decrease the power density to 17.48 mW/cm^2^, which can be attributed to the rise in resistance components such as R_s_, R_ct_, and R_t_ within the thicker IED. This conclusion can be proved by the EIS analysis (Figure S5A) and polarization slope of the linear part of the current (I) vs. voltage (V) plot (Figure 4C): The R_t_ were 14.64, 24.81, and 39.63 Ω for the IED of 0.15, 0.30, and 0.45 mm, respectively; R_s_ and R_ct_ also display consistent trends in their variations, as detailed in Table S7. Despite the presence of current reversal in the 0.15 mm thick IED in the low‐pressure region, the thinner IED offers advantages in terms of overall performance, possibly due to its reduced resistance to ion transport and improved charge transfer efficiency. Through systematic modulation of IED (0.15–0.45 mm, Δ = 150 mm intervals) coupled with comprehensive performance evaluation (polarization curves), this study establishes an optimal compromise between interfacial charge transfer kinetics (favored by thinner layers) and bulk electrolyte retention capacity (enhanced by thicker structures), ultimately identifying 0.15 mm as the critical thickness.

### 2.5 Electrolyte Concentrations

**FIGURE 5:**
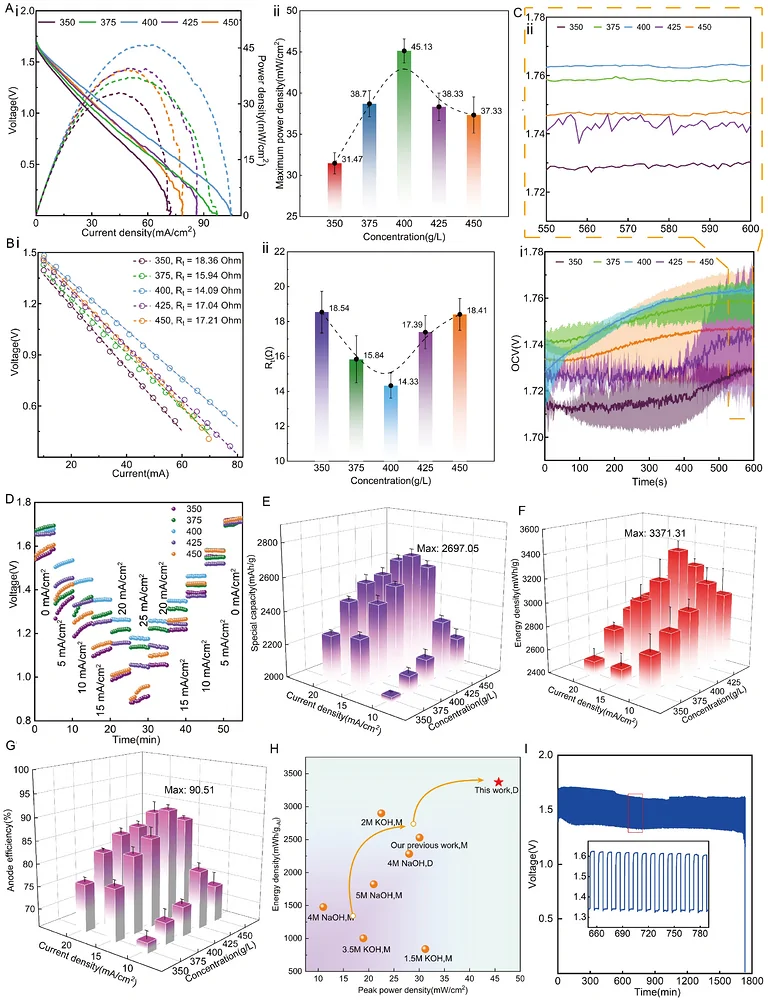
Performance of D‐MFAAB with different electrolyte concentrations. (A) Power density curves. (B) Polarization curves. (C) OCV. (D) U‐I characteristics at 0, 5, 15, 20, 25, 20, 15, 10,5 and 5 mA/cm^2^. (E) Galvanostatic performance at 10 mA/cm^2^. (F) Specific capacity. (G) Energy density. (H) Comparison of power density vs. energy density of D‐MFAABs with other energy storage devices. (I) The On/off alternate test of D‐MFAAB with 400 g/L.

Figure 5 shows the effect of the Electrolyte concentrations on the performance of D‐MFAAB. The concentrations are 350, 375, 400, 425, and 450 g/L, respectively. As shown in Figure 5A, increasing the electrolyte concentration from 350 to 400 g/L boosted the maximum power density by 38.7% (31.47 → 45.13 mW/cm^2^) through accelerated Al anode corrosion, which reduced activation polarization and elevated output voltage [33]. In addition, the increased electrolyte concentration also optimized the charge transfer between the separator, Al anode, and air cathode, reducing R_t_ from 18.36 (350 g/L) to 14.33 Ω  (40 g/L) (Figure 5B and Table S1).

Over concentrated electrolyte hampers the peak power density from 45.73 to 37.33 mW/cm^2^ of 450 g/L. While electrolyte concentrations beyond a critical threshold (>400 g/L) may retain sufficient ionic conductivity, such hyperalkaline conditions accelerate cathode catalyst corrosion through two synergistic pathways: (1) oxidative dissolution of transition metal species and (2) chemical decomposition of cellulose‐based separators via elimination reactions, ultimately leading to membrane pore collapse and catalyst delamination [40]. Furthermore, the increased viscosity of highly concentrated electrolytes reduces the diffusivity of hydroxide ions, which may counteract the positive effects associated with higher OH^−^ concentration. As shown in Figure 5B and Table S1, there is no significant difference for R_s_ of different electrolyte concentrations, but both R_ct_ and R_t_ reach their respective troughs at approximately 400 g/L, after which they both exhibit an increasing trend. The results indicate that using an excessively high concentration of electrolytes is bad battery operation stability and can inhibit the generation of power.

As depicted in Figure 5C, the OCV exhibits significant concentration dependence. The 400 g/L electrolyte system demonstrates the highest and most stable OCV of 1.762 V, compared to 350 g/L (1.73 V) and 450 g/L (1.73 V) counterparts. This optimal behavior stems from synergistic effects between ionic mobility enhancement (R_t_ = 14.09 Ω), as quantified by EIS (Figure 1F) and linear polarization resistance analyses (Figure 5B). At the current density of 0, 5, 10, 15, 20, 25, 20, 15, 10, 5, and 0 mA/cm^2^, the discharge voltage of the battery exhibits a parallel trend with power, initially rising and subsequently declining gradually with the increase of electrolyte concentration (Figure 5D). The galvanostatic discharge performance of the batteries using various electrolyte concentrations at current densities of 10, 15, and 20 mA/cm^2^ was tested. Although the battery discharge duration is different for each current density, the same regularity is indicated. Aluminum–air batteries are typical primary batteries that operate via the oxidation of the aluminum anode. In contrast to lithium‐ion batteries, where the voltage gradually declines during discharge, the discharge voltage of aluminum–air batteries remains largely stable as long as the internal chemical environment does not undergo significant changes. Once the aluminum anode is completely consumed, however, the voltage collapses abruptly. This behavior accounts for the feature observed in the polarization curve, where the voltage initially remains stable and then drops sharply over time. The average discharge voltage increases with the increase of electrolyte concentrations and decreases when it exceeds 400 g/L. At the same current density, the battery discharge duration also follows the above regularity. Finally, the specific capacity Q, anode efficiency 𝜂, and energy density W of the battery at 10, 15, and 20 mA/cm^2^ are shown in, Figure 5E–G, respectively. At 15 mA/cm^2^, the Al‐air battery with the electrolyte concentration of 400 g/L achieves maximum specific capacity and anode efficiency, about 2697.05 mAh/g_Al_ and 90.51%, respectively. Besides, a high energy density of 3371.31 mWh/g_Al_ is also obtained. Therefore, 400 g/L is considered the optimal concentration to achieve maximizing battery discharge performance and activation of Al anode simultaneously. In addition, Figure 5H presents a comparison of the specific capacity and energy density of recently reported‐ AABs with disparate electrolyte storage methodologies. Among similar battery designs, our D‐MFAAB demonstrates the most superior performance [24, 25, 26, 27, 28, 29, 36, 37, 38, 40, 46, 47, 49, 50, 51, 52, 53, 54, 55], laying a solid foundation for its practical application in the next phase. In Figure 5I, an on/off alternate test (10 mA/cm^2^) was performed to simulate the practical application of AABs. The OCV and the working potential were 1.71 and 1.37 V, respectively, and the battery shows a long lifespan of 29.4 h.

### 2.6 Application of D‐MFAAB for Miniaturized Devices and Miniaturized Biomorphic Robots

**FIGURE 6:**
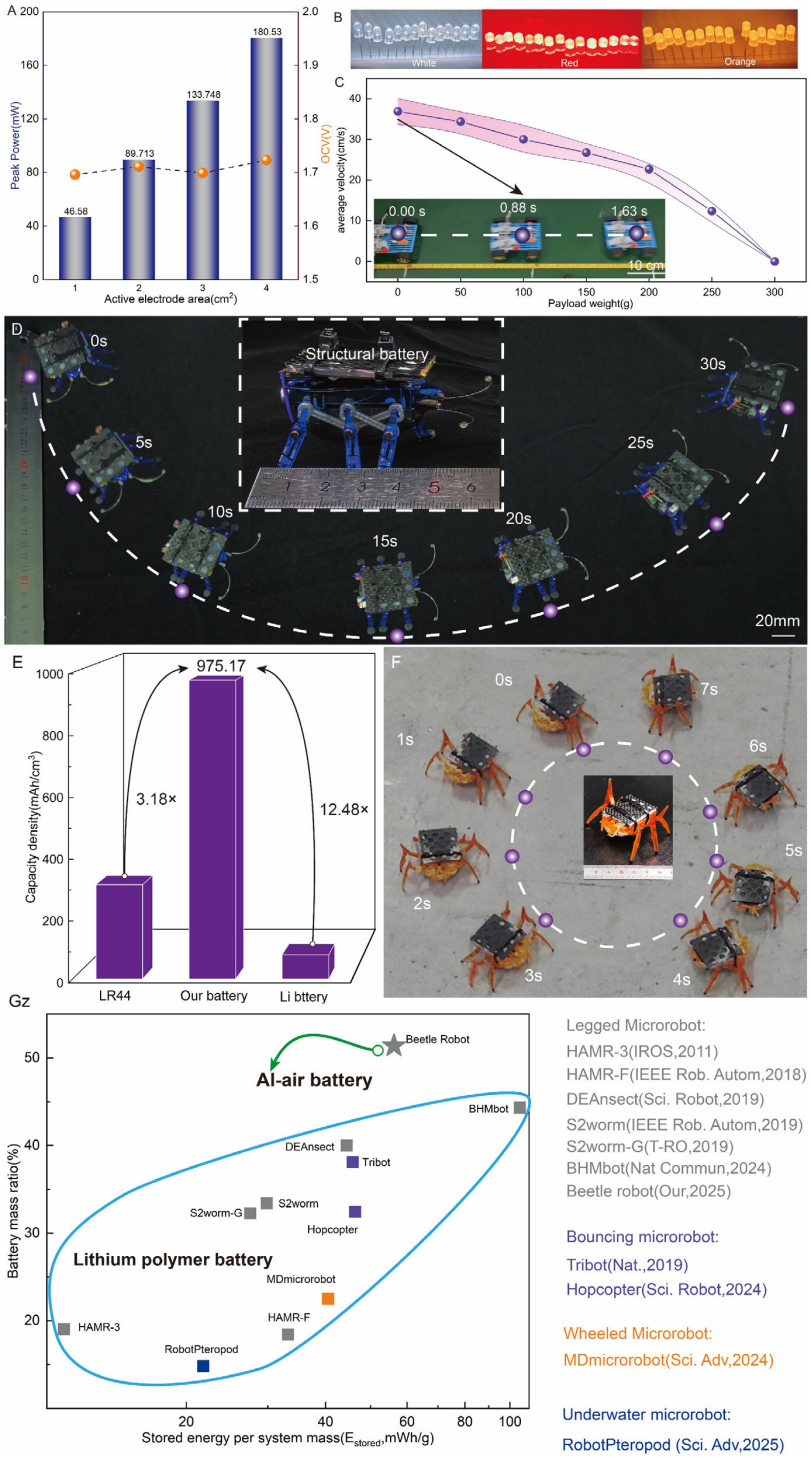
Applications of D‐MFAABs. (A) The peak power of D‐MFAAB with active area of 1, 2, 3, and 4 cm^2^. (B) and (C) Two series‐connected D‐MFAABs powering various‐color LED lamps and an electric toy car. (D) The Beetle Robot crawls on both ordinary ground and plastic sealing film surfaces. (E) The volumetric capacity density of three batteries for miniature robots. (F) The Crab Robot crawls on floor glue. (G) Comparison of the battery mass ratio and energy storage in microrobots.

A single D‐MFAAB with the active area of 1 cm^2^ can only provide an OCV of 1.70 V and the power of 45.69 mW, which is not sufficient for most applications. Initially, three approaches were employed to enhance the output power using a 2 cm^2^ active area: configuring cells in series or parallel connections, and directly increasing the electrode size. Figure S6 illustrates the polarization curves for above 3 methods, the method of directly increasing the electrode area demonstrated superior performance compared to series connection and parallel connection, achieving the power of 89.73 mW. This enhanced performance is attributed to low resistance for larger area of a single electrode. Therefore, we focused on directly increasing the electrode area to enhance the D‐MFAAB output power. As the electrode area increases, the battery output power increases almost linearly, with an average increase of approximately 45 mW for every additional square centimeter, demonstrated in Figure 6A. However, this method also introduces drawbacks: the OCV maintains a near‐constant value with the electrode area extension.

Battery encapsulation was implemented using lightweight carbon fiber plates, flexible polyimide (PI) film hinges, and adhesive, with electrolyte concentration maintained at 400 g/L throughout experiments unless specifically modified. The battery active area is 0.5 cm × 1 cm × 2, similar to a coin. With the adequate and smooth supply of electrolyte in the separator, a single battery is sufficient to illuminate white, red, orange, and blue LEDs (Figure 6B). Additionally, a D‐MFAAB drive a DC motor (Figure S7A) or a mini‐fan (Figure S7B). Owing to the lightweight nature and high energy density of Al, D‐MFAAB is suitable for applications in electric vehicles. To study the ability of the D‐MFAAB to provide power for a moving machine, a D‐MFAAB stack, consisting of two 0.5 cm × 1 cm × 2 cells connected in series, was created. The stack was sufficient to drive the electric toy car in a straight line with a speed of 36.88 cm/s (Figure 6C). Figure 6C also shows how increasing the cargo mass affected the vehicle velocity. As the payload mass was increased from 50 to 250 g, the velocity decreased from 34.37 to 12.36 cm/s (Movie S1).

Our battery employs carbon fiber plate clamping technology to complete the encapsulation process, ranting the battery exceptional stiffness characteristics to provide new design perspective for robotic. Both the electrode and separator of the battery exhibit deformation tolerance, thereby facilitating a multifunctional design for body‐integrated energy storage batteries. We replaced the original button batteries (LR44) in the small‐scale robots' torso with a one‐dimensional SEES, which simultaneously provides energy storage and mechanical load‐bearing functions, as demonstrated in Figure 6D. Experimental results demonstrate significant improvements in operational endurance and volumetric energy density. When powered by two LR44 button cells, the robotic system exhibited an operational duration of approximately 130 min. In contrast, the structural battery (RSB) extended continuous operation to 240 min, achieving a 100% enhancement in energy sustainability. Quantitative analysis of volumetric capacity reveals the superior performance of the bio‐inspired energy system (SEES). Through volumetric energy density normalization, SEES demonstrates 3.18× and 12.48× improvements over conventional LR44 button cells and lithium‐polymer batteries (401015), respectively, showing in Figure 6E and Supplementary Information. This density advantage confirms the system's exceptional spatial energy efficiency, particularly critical for size‐constrained robotic applications. A practical robot must be capable of navigating across diverse terrains, climbing slopes, and carrying payloads. The first is crawling capabilities of the robot on surfaces with different levels of roughness. The Beetle Robot demonstrates consistent performance when operating on flat terrain: moving on both ordinary ground (Figure S7C) and plastic sealing film surfaces (Figure S7D and Movie S2). Robotic slope‐climbing performance was systematically evaluated through inclined plane tests (θ = 8.39°) using standardized wooden ramps (15.0 × 35.5 cm^2^), where the Beetle Robot exhibited 2.36 cm/s ascent velocity, as detailed in Figure S7E and Movie S3. The Crab Robot also possesses similar locomotor capabilities (Figure 6F; Figure S7F and Movie S4). Figure 6G compares the battery mass ratio and energy storage per system mass among legged microrobots, jumping microrobots, underwater microrobots, wheeled microrobots powered by Li‐polymer batteries, and our self‐sustained robot powered by an Al‐air battery. This comparison demonstrates that our SEES mechanism enables the microrobot to maintain a relatively compact body size while achieving an energy storage density (55 mWh/g) that surpasses most lithium‐ion battery solutions. For more intuitive comparison, Table 2 summarizes the performance of the aforementioned microrobots and our prototype across five key metrics (i.e., body mass, onboard energy, operation duration, stored energy per system mass, and battery mass ratio). The comparison demonstrates superior performance in both operation duration and onboard energy, while maintaining body mass and battery mass ratio comparable to those of other robotic platforms. Although the battery accounts for over 50% of the total mass, this should be considered in the context of multifunctional design: the battery simultaneously serves as a structural component, mitigating the ‘dead weight’ penalty. For insect‐inspired robots with minimal payload requirements, this trade‐off enables sustained operation without compromising mechanical integrity.

**TABLE 2:** Five key metrics of microrobots.

Microrobot	Mass (g)	Energy (mWh)	Operating Time (min)	Stored energy per system mass (mWh/g)	battery mass ratio (%)
**Beetle Robot (Our robot)**	**14.21**	**798.7**	**240.0**	**56.21**	**51.38**
HAMR‐F [56]	2.79	92.5	4.5	33.15	18.44
HAMR‐3 [57]	1.70	18.5	18.5	10.88	19.06
DEAnsect [21]	1.00	44.4	14.0	44.40	40.00
S^2^worm [58]	4.34	129.5	13.0	29.84	33.41
S^2^worm‐G [59]	4.71	129.5	14.0	27.49	32.27
Tribot [60]	9.70	444.0	16.0	45.77	38.14
BHMbot [8]	1.76	185.0	3.0	105.11	44.32
RobotPteropod [2]	34.00	740.0	76.0	21.76	14.82
Hopcopter [61]	51.80	2400.0	50.9	46.33	32.43
MDmicrorobot [1]	6.40	259.0	259.0	40.47	22.50

## 3 Discussion

Embodied energy design represents a promising approach to address the endurance limitations of insect‐scale robots, as it integrates both energy storage and mechanical functions within a single structure, maximizing energy density within constrained volumes. This concept has been successfully implemented and validated in larger robotic platforms [62, 63, 64], demonstrating significantly enhanced operational endurance compared to conventional lithium‐ion battery solutions. Concurrently, the development of high‐energy‐density batteries offers a complementary pathway to further improve robot endurance. We demonstrate, for the first time, an insect‐scale robot powered by a high‐energy‐density aluminum‐air battery system integrated via embodied energy storage design, achieving sustained locomotion. This robot exhibits an operational endurance of approximately 4 h, showcasing a substantial improvement in untethered runtime.

However, the operational lifespan and stored energy per system mass of the robot was significantly lower than its theoretical limit. During the experiment, electrolyte was replenished only once. The continuous decrease in electrolyte concentration during discharge led to a decline in power density. Post‐experiment analysis revealed substantial unconsumed aluminum anode material, indicating a mismatch between anode consumption rate and electrolyte reservoir capacity in the current design, which constrained the overall system performance. Future enhancements could focus on: (i) optimizing the aluminum anode properties (e.g., reducing thickness, employing mesh structures); (ii) refining the battery design (e.g., utilizing separators with superior electrolyte retention and higher ionic conductivity); and (iii) improving the robot structure (e.g., incorporating miniaturized electrolyte reservoirs). These modifications are expected to enhance battery performance and accelerate the discharge kinetics.

Inspired by organisms acquiring sustenance from their environment to sustain life, exploring the deep integration of metal‐air batteries with insect‐scale robotic structures presents a promising pathway toward constructing a revolutionary energy harvesting and replenishment system. This approach holds the potential to achieve breakthrough enhancements in robot energy storage density. Concurrent structural innovations facilitate rapid metallic anode exchange, allowing mechanical “recharging” of electrodes at energy flow rates comparable to refueling internal combustion engines. Such insect‐scale robots, endowed with “active energy ingestion” capabilities—effectively enabling direct “metabolism” of high‐energy‐density fuels—could dramatically reduce or even eliminate dependence on traditional chemical batteries. We believe this paradigm not only imbues robots with unprecedented operational “vitality” but also promises to significantly augment their autonomy and environmental resilience. Consequently, these systems could sustain prolonged, high‐endurance operations in complex, remote, or extreme environments, thereby paving the way for a new era of autonomous robotic applications.

## 4 Materials and Methods

### 4.1 Materials

Al_5N_ (Al purity:99.999%) was purchased from Wenzhou Metal Materials Business Co. Ltd. (China). Al_1N_ (Al purity:97.645%) was purchased from Coca‐Cola Beverages Co. Ltd. (China). Al_3N_ electrodes (Al purity: 99.900%) were purchased from Hunan Seirios New Material Technology Co. Ltd. (China). The exhaustive compositions of the three aluminum anodes are comprehensively outlined in Table S8. Hunan Seirios New Material Technology Co., Ltd. (located in Changsha, China) has friendly provided air cathodes, whose uniqueness lies in the ingenious fusion of the catalytic layer, collector layer, and waterproof layer into a single, integrated component. In addition, general clean wiper (GCP) (manufacturer: Sangon Biotech (Shanghai) Co., Ltd., China), qualitative filter paper (WR) (manufacturer: Hangzhou Fuyang Beimu Pulp Paper Co., Ltd., China), and kitchen paper (JR) (manufacturer: C&S Paper Co., Ltd., China) were used as separator and micro‐channel for Al‐air battery.

### 4.2 Electrolyte Preparation Method

Weigh a certain amount of solid‐state electrolyte powder according to the set polarization, dissolve it in deionized water to form a mixed solution. After the solution returns to room temperature, transfer it to a bottle. Add an appropriate amount of deionized water to obtain an electrolyte with the corresponding concentration (g/L).

### 4.3 Battery Characterization

Upon completion of the battery assembly, the electrolyte naturally migrates to the power‐generating core of the battery, guided by the separator's capillary action and gravitational effect, effectively initiating the battery's activation process. Once it reaches a stable equilibrium in the potential difference between the anode and cathode, the open‐circuit voltage (OCV) will be recorded. The battery polarization and power curve were gained by linear sweep voltammetry (LSV), from OCV to 0 V with a scan rate of 5 mV/s, and the power density was calculated by Equation (7) and normalized to the active geometric area of the electrode A (1 cm × 1 cm, or 0.5 cm × 1 cm × 2). The electrochemical impedance spectroscopy (EIS) test was performed with an amplitude of 10 mV and a frequency range of 10^5^–0.1 Hz at OCV. The solution resistance (R_s_) and charge transfer resistance (R_ct_) were determined from the equivalent circuit from fitted EIS spectra. The total resistance (R_t_) of batteries was calculated by Equation (8) with the polarization slope method, utilizing the linear part of the current (I) vs. voltage (V) plot. All electrochemical tests were performed at least three times using an electrochemical workstation (DH7000C, Donghua Co. Ltd., China) at room temperature, and the dataset with the average value of the observed indicator was selected for plotting.

(7) $$P_{d}=\frac{U\times I}{A}$$

(8) $$R_{t}=\left|\frac{dU}{dI}\right|=\frac{-\Delta U}{\Delta I}$$

### 4.4 Performance Evolution

A multi‐channel battery test system (LAND CT3002A, manufactured by Wuhan LAND Electronic Co., Ltd, China) is utilized to evaluate the discharge characteristics of the fully assembled battery. First, monitor and record the open circuit voltage (OCV) of batteries with varying concentrations of electrolyte over 10 min. Next, the U‐I characteristic was tested at the current densities of 0, 5, 10, 15, 20, 25, 20, 15, 10, 5, and 0 mA/cm^2^ with a time step of 5 min. Then, the battery discharge experiments at I = 10, 15, and 20 mA/cm^2^ were carried out, respectively, and the change of voltage U (V) with time T (h) was also recorded. The specific capacity (Q, mAh/g_Al_) and utilization efficiency (𝜂, %) of the Al anode and energy density (W, mWh/g_Al_) of the battery at different current densities were calculated, respectively, based on the consumption of Al (△m_Al_, g), as outlined in Equations (9)–(11). Besides, the on/off cyclic discharge performance is also explored, with each cycle consisting of 5‐min discharge followed by 5 min‐rest at the operating currents of 10 mA/cm^2^.

(9) $$Q=\frac{\int_{0}^{T}Idt}{\Delta m_{Al}}=\frac{IT}{\Delta m_{Al}}$$

(10) $$\eta =\frac{Q}{Q_{0}}\times 100\%$$

(11) $$W=\frac{\int_{0}^{T}UIdt}{\Delta m_{Al}}=\frac{I\int_{0}^{T}Udt}{\Delta m_{Al}}=\frac{IU_{e}T}{\Delta m_{Al}}$$

where I, T, and U were the discharge current, time, and the voltage, respectively. U_e_ is the average discharge voltage over T time at the discharge current density I. Q_0_ is the theoretical specific capacity of aluminum (2980 mAh/g_Al_).

### 4.5 Battery Assembly

This study involves three aluminum‐air battery systems with structural heterogeneity, all sharing a unified air cathode fabrication process: Catalysts are uniformly compounded with polymer binders and coated onto nickel mesh current collectors to form integrated functional electrodes, effectively circumventing the need for additional integration of current collectors in traditional battery designs. The structural characteristics and evolutionary relationships of each system are outlined below:

1. M‐MFAAB: Adopting a classic sandwich configuration, it features a laminated structure sequentially composed of an aluminum‐based anode, polymer separator, and air cathode. To optimize electrical contact performance and maintain structural stability, a polyimide (PI) film‐hinged carbon fiber backing plate is introduced as external support during battery encapsulation. Precision‐engineered arrayed through‐hole structures on the backing plate ensure effective mass transfer interfaces between the cathode active layer and atmospheric medium.
2. D‐MFAAB: Building upon the M‐MFAAB, topological optimization is implemented through a dual‐reactive‐surface aluminum anode, constructing a symmetric five‐layer architecture: air cathode || separator || aluminum anode (dual‐active surfaces) || separator || air cathode. This structural innovation achieves approximately 100% improvement in anode material utilization while balancing internal stress distribution via symmetric electrode arrangement.
3. SEES: Leveraging the structural features of D‐MFAAB, biomimetic morphology design is performed in alignment with the geometric characteristics of insect‐scale robot platforms. The system integrates the battery architecture with robotic actuation mechanisms through conformal engineering, ensuring spatial compatibility between SEES and robotic systems while preserving electrochemical performance.

### 4.6 Battery Activation and Electrolyte Priming Procedure

The microfluidic configuration employed in this study relies on capillary force to drive electrolyte transport. The battery activation procedure was performed as follows:

#### 4.6.1 Pre‐Wetting Treatment

Prior to assembly, the cellulose separator was cut to the appropriate dimensions and immersed in deionized water for 10 s to remove air bubbles and enhance hydrophilicity. After removal, excess surface water was gently blotted with filter paper, and the separator was immediately incorporated into the battery stack.

#### 4.6.2 Electrolyte Priming

After assembly and clamping, one end of the microfluidic channel was immersed in an electrolyte‑containing reservoir through a small aperture to ensure direct contact with the electrolyte. Driven by capillary action, the electrolyte was transported through the microchannel and spontaneously wetted the entire electrode region. The movement of the electrolyte front could be visualized by adding a trace amount of dye to the solution.

#### 4.6.3 Stabilization Criteria

Following complete wetting, the open‑circuit voltage (OCV) gradually increased from its initial value and stabilized at 1.7–1.9 V within 10 min. When the OCV fluctuation remained below ±0.05 V for more than 1 min, the battery was considered to have reached a stable operating state and was ready for discharge testing.

#### 4.6.4 Intermittent use and Re‑Priming

➢ Short‑term interruption (< 30 min): If the battery was idle for a short period and the microchannel remained moist, it could proceed directly to the next test without re‑priming.
➢ Prolonged interruption or drying: If the battery was idle for an extended period or the microchannel had dried out, electrolyte re‑priming or replacement of the microfluidic channel was required following the same procedure described above.

#### 4.6.5 Electrolyte Replenishment

During prolonged continuous discharge, if a voltage drop was observed and electrolyte depletion was confirmed, fresh electrolyte was injected into the electrolyte reservoir using a syringe. The voltage typically recovered to its original level within a short time after replenishment.

### 4.7 Integration of SEES for Miniature Robots

The hexapod biomimetic robot and crab‐type robot prototypes were procured from the Tmall platform. To achieve structural‐functional integration between the SEES (Bio‐inspired Integrated Energy System) and the robotic platforms, we systematically replaced the original protective structures with the SEES through compatibility‐optimized design, serving as their protective enclosure. The outer casing of the energy system employs a flexible porous carbon fiber composite, insulated via dielectric bonding process to mitigate electrochemical short‐circuit risks. The SEES architecture incorporates a modular design philosophy from the conceptual phase, enabling its electrode stacking units to achieve series/parallel topological reconfiguration through standardized interfaces, which demonstrated exceptional scalability. In all experimental configurations, the SEES successfully realized dual‐functional integration: (1) Acting as a mechanical protective layer for the robotic chassis; (2) Replacing conventional standalone power systems to accomplish energy‐structure integration. This innovation establishes a paradigm where energy storage components concurrently fulfill structural and protective roles, demonstrating cross‐scale adaptability from macro rigid robots to micro flexible robotic devices.

## Author Contributions

Y.Y., T.J., Z.L., and D.F. conceived and conceptualized the study. The methodology was developed by Y.Y. and Z.Y. Investigation and data collection were performed by Y.Y. and Z.L. Experiments, data analysis, and visualisations were conducted by Y.Y. and T.J. Y.Y. wrote the original draft of the manuscript. Review and editing of the manuscript were carried out by Y.Y., T.J., Z.L., and D.F. Funding acquisition was secured by T.J. and Z.L.

## Funding

This work was financially supported by the National Natural Science Foundation of China (52575351), Excellent Young Scientists Fund of Hunan Province of China (2024JJ4043), the Cornerstone Capital project of the National University of Defense Technology (JS202306), and Hunan Provincial Innovation Foundation for Postgraduaten (CX202500094).

## Conflicts of Interest

The authors declare no conflicts of interest.

## Supporting information




**Supporting File 1**: advs75702‐sup‐0001‐SuppMat.docx.

**Supporting File 2**: advs75702‐sup‐0002‐MovieS1‐S4.zip.

## Data Availability

The data that supports the findings of this study are available in the supplementary material of this article.
