# Supplementary material for: An Embodied Paper‐Based Microfluidic Al‐Air Battery for Enduring Untethered Insect‐Scale Robots
Source: Adv Sci (Weinh). 2026 May 30;13(45):e75702. doi: 10.1002/advs.75702 (PMC13336105; doi:10.1002/advs.75702)
Supplement: Supplementary file 1 [file ADVS-13-e75702-s001.docx]

Supplementary Materials for

**An embodied paper-based microfluidic Al-air battery for enduring untethered insect-scale robots**

Yun Yang *et al.*

*Corresponding author. Email: [jiangtao@nudt.edu.cn](mailto:jiangtao@nudt.edu.cn), [luozirong@nudt.edu.cn](mailto:luozirong@nudt.edu.cn)

**Figure S1** **A** Power density benchmark: conventional electrolyte vs modified electrolyte. **B** High-frequency short semicircle radius for EIS. **C** Anode/cathode Single electrode polarization.

**Figure S2** Comparison of discharge performance between D-MFAAB and M-MFAAB

**Figure S3** Electrochemical performance of batteries with different anode materials

**Figure S4** EIS characteristics and electrolyte retention capacity of different separator materials

**Figure S5** Effect of inter-electrode distance on electrochemical performance of batteries

**Figure S6** Constant-current discharge performance of D-MFAAB at RT.

**Figure S7** Demonstration of D-MFAAB in low-power devices and SEES.

**Table S1** Interface resistance information of self-filtering Al-air batteries under different operation conditions

| No. | **electrolyte concentration**  (g L^-1^) | Anode materials | Separators | *R_s_*  (Ω) | *R_1_*  (Ω) | *R_2_*  (Ω) | *R_3_*  (Ω) | *R_ct_*  (Ω) | *R_t_*  (Ω) |
| --- | --- | --- | --- | --- | --- | --- | --- | --- | --- |
| 1 | 350 | Al_3N_ | GCP | 0.58 | 4.74 | 28.14 | 6.40 | 4.74 | 18.36 |
| 2 | 375 |  |  | 0.71 | 4.81 | 2.341 | 69.43 | 2.31 | 15.94 |
| 3 | 400 |  |  | 0.59 | 1.063 | 5.50 | 123.8 | 1.06 | 14.09 |
| 4 | 425 |  |  | 0.76 | 0.61 | 6.63 | 36.25 | 0.61 | 17.04 |
| 5 | 450 |  |  | 1.67 | 7.62 | 0.14 | 54.13 | 1.67 | 17.21 |

**Table S2** Calculation parameters and results of relative texture coefficient (RTC) of the Al1N, Al3N and Al5N anodes at initial state. The relative intensities of standard diffraction I0(hkl) are from PDF-#85-1327.

| Intensity | I_(111)_ | I_(200)_ | I_(220)_ | I_(311)_ |
| --- | --- | --- | --- | --- |
| I_0_ | 100 | 47 | 22 | 24 |
| Al_1N_ | 2029 | 24009 | 19839 | 17017 |
| Al_3N_ | 18297 | 73059 | 25941 | 7293 |
| Al_5N_ | 7552 | 121161 | 84306 | 34943 |

**Table S3** EIS fitting results of different anodes in different temperature

| Smaple | Temperature℃ | Rct  Ω | Smaple | Temperature℃ | Rct  Ω | Smaple | Temperature  ℃ | Rct  Ω |
| --- | --- | --- | --- | --- | --- | --- | --- | --- |
| Al_1N_ | 20 | 0.5979 | Al_3N_ | 20 | 0.2089 | Al_5N_ | 20 | 0.4029 |
|  | 30 | 0.4659 |  | 30 | 0.1607 |  | 30 | 0.2225 |
|  | 40 | 0.2146 |  | 40 | 0.0914 |  | 40 | 0.1500 |
|  | 50 | 0.1487 |  | 50 | 0.0575 |  | 50 | 0.1003 |
|  | 60 | 0.0939 |  | 60 | 0.0539 |  | 60 | 0.0670 |

**Table S4** The EIS fitting results for assembled battery with **different anodes**

| No. | electrolyte concentration  (g L^-1^) | **Anode materials** | Separators | *R_s_*  (Ω) | *R_1_*  (Ω) | *R_2_*  (Ω) | *R_3_*  (Ω) | *R_ct_*  (Ω) | *R_t_*  (Ω) |
| --- | --- | --- | --- | --- | --- | --- | --- | --- | --- |
| 1 | 400 | Al_1N_ | GCP | 0.52 | 1.08 | 2.03 | 69.52 | 1.08 | 14.36 |
| 3 |  | Al_3N_ |  | 0.59 | 1.063 | 5.50 | 123.8 | 1.063 | 14.00 |
| 55 |  | Al_5N_ |  | 1.67 | 7.62 | 0.14 | 54.13 | 7.62 | 12.74 |

**Table S5** The EIS fitting results for assembled battery with **different** separator materials

| No. | electrolyte concentration  (g L^-1^) | Anode materials | **Separators** | *R_s_*  (Ω) | *R_1_*  (Ω) | *R_2_*  (Ω) | *R_3_*  (Ω) | *R_ct_*  (Ω) | *R_t_*  (Ω) |
| --- | --- | --- | --- | --- | --- | --- | --- | --- | --- |
| 1 | 400 | Al_3N_ | GCP | 0.58 | 4.74 | 28.14 | 6.40 | 4.74 | 14.00 |
| 2 |  |  | JR | 0.60 | 1.97 | 8.86 | 45.72 | 1.97 | 21.99 |
| 3 |  |  | WR | 0.56 | 4.49 | 0.83 | 67.91 | 4.49 | 14.19 |

**Table S6** Water retention capacity testing of different diaphragm materials

| Type | Num. | M_0_ (g) | M_1_ (g) | $\Delta\text{M}$ (g) | K | Avg |
| --- | --- | --- | --- | --- | --- | --- |
| WR | 1 | 0.431 | 7.474 | 7.043 | 9.38 | 9.07 |
|  | 2 | 0.462 | 4.588 | 4.126 | 8.93 |  |
|  | 3 | 0.465 | 4.603 | 4.138 | 8.9 |  |
| JR | 1 | 0.373 | 3.314 | 2.941 | 7.88 | 7.94 |
|  | 2 | 0.399 | 2.58 | 2.181 | 7.97 |  |
|  | 3 | 0.428 | 3.835 | 3.407 | 7.96 |  |
| GCP | 1 | 0.278 | 2.701 | 2.423 | 8.72 | 9.24 |
|  | 2 | 0.265 | 2.591 | 2.326 | 8.78 |  |
|  | 3 | 0.262 | 2.824 | 2.562 | 9.78 |  |
|  | 4 | 0.276 | 2.814 | 2.538 | 9.69 |  |

**Table S7** The EIS fitting results for assembled battery with **different** inter-electrode distance

| No. | **inter-electrode distance**  (mm) | electrolyte concentration  (g L^-1^) | Anode materials | Separators | *R_s_*  (Ω) | *R_1_*  (Ω) | *R_2_*  (Ω) | *R_ct_*  (Ω) | *R_t_*  (Ω) |
| --- | --- | --- | --- | --- | --- | --- | --- | --- | --- |
| 1 | 0.15 | 400 | Al_3N_ | GCP | 0.59 | 1.06 | 5.50 | 1.06 | 14.64 |
| 2 | 0.30 |  |  |  | 0.57 | 2.38 | 31.13 | 2.38 | 24.81 |
| 3 | 0.45 |  |  |  | 0.60 | 4.29 | 63.09 | 4.29 | 39.63 |

**Table S8** Chemical composition and Al anode used in Al-air batteries

| Type of Al anode | Al (%) | Si (%) | P(%) | S(%) | Mg(%) | Zn(%) | Ti(%) | Fe(%) | Cu(%) | Cr(%) |
| --- | --- | --- | --- | --- | --- | --- | --- | --- | --- | --- |
| Al_3N_ | 99.9000 | 0.0758 | 0.0266 | 0.0160 | — | — | — | — | — | — |
| Al_1N_ | 97.6450 | 0.4000 | — | — | 0.8000 | 0.2500 | 0.0150 | 0.7000 | 0.1500 | 0.0400 |
| Al_5N_ | 99.9990 | — | — | — | — | — | — | — | — | — |

Note: the chemical composition of Al anodes were obtained by EDS analysis.

Supplementary Text

The SEES exhibits the following key characteristics: an effective working area of 2 × 2 cm², with the aluminum anode mass calculated as 0.909 g based on its areal density. When integrated into the robotic structural-power module, the system demonstrates a compact packaged volume of 2.89 cm³. Through volumetric normalization analysis, the engineered configuration achieves exceptional performance metrics, delivering a calculated volumetric capacity of 975.15 mAh/cm³. Considering the parameters of the original LR44 battery (1.5 V, 175 mAh), we estimate that the total charge of the SEES (2451.62 mAh) replacing the protective covers will be 7 times higher than the current stand-alone LR44 battery. For comparative purposes, the capacity was normalized to 307 mAh/cm³ for comparative analysis (approximately 30% of the SEES benchmark value).

The volume of the Commercial lithium polymer battery (401016) is V=0.575 cm^3^; the stored charge is 45 /0.575 =78.26 mAh/cm^3^ (approximately 8.01% of the SEES benchmark value).

**Safety and Environmental Considerations**

The electrolyte used in this study is strongly alkaline (pH > 14) and contains fluoride ions, necessitating careful handling and disposal. In laboratory settings, we implemented the following protective measures: (1) operators wear alkali-resistant gloves, goggles, and lab coats; (2) battery assembly is conducted in a fume hood; (3) dilute acetic acid solution is kept on hand as a neutralizing agent in case of accidental spillage.

In the event of leakage during robot operation, the strongly alkaline electrolyte could corrode structural components or circuits and contaminate the operating environment. The presence of fluoride further increases environmental concerns, as free fluoride ions may impact soil and water systems. Therefore, spent batteries should be treated as hazardous chemical waste and must not be discarded indiscriminately. A recommended disposal procedure includes: first, diluting the residual electrolyte with copious water and neutralizing to pH 7, then discharging according to local environmental regulations; the consumed aluminum anodes and separators can be collected as solid waste for recycling.

For future practical applications, we propose the following improvements: (1) developing gel or solid-state electrolytes to fundamentally eliminate leakage risks; (2) employing double-layer encapsulation or anti-corrosion coatings to protect the robot body; (3) designing electrolyte circulation or real-time monitoring systems to provide early warning before electrolyte depletion, thereby preventing leakage.

$$4Al^{3+}+6F^{-}+12OH^{-}\to AlF_{6}^{3-}+3Al(OH)_{4}^{-}$$

To calculate the change in Gibbs free energy ($\Delta_{r} G^{\circ}$) for this reaction, the most commonly used approach is to employ the standard molar Gibbs free energy of formation ($\Delta_{f} G^{\circ}$). The formula is:

$$\Delta_{r} G^{\circ}=\sum\nu_{i} \Delta_{f} G^{\circ}\text{(Products)}-\sum\nu_{j }\Delta_{f} G^{\circ}\text{(Reactants)}$$

where $\nu_{i}$ and $\nu_{j }$ are the stoichiometric coefficients of the products and reactants, respectively.

Standard molar gibbs free energy of formation for each species (at 298.15 K). Obtained from thermodynamic data tables :

$$\Delta_{f} G^{\circ}_{\text{Al}^{\text{3+}}(aq)}=-489.4kJ\cdot mol^{-1}$$

$$\Delta_{f} G^{\circ}_{\text{F}^{-}(aq)}=-278.8kJ\cdot mol^{-1}$$

$$\Delta_{f} G^{\circ}_{\text{OH}^{-}(aq)}=-157.24kJ\cdot mol^{-1}$$

$$\Delta_{f} G^{\circ}_{\text{Al}F_{6}^{3-}(aq)}\approx-2056.0kJ\cdot mol^{-1}$$

$$\Delta_{f} G^{\circ}_{\text{[Al(OH)}_{4}]^{-}(aq)}=-1305.0kJ\cdot mol^{-1}$$

$$\begin{matrix} \sum\nu_{i} \Delta_{f} G^{\circ}\text{(Products)=}\Delta_{f} G^{\circ}_{\text{Al}F_{6}^{3-}(aq)}+3\times\Delta_{f} G^{\circ}_{\text{[Al(OH)}_{4}]^{-}(aq)} \\ \text{=}-2056.0-3\times1305.0=-5971.0kJ\cdot mol^{-1} \end{matrix}$$

$$\begin{matrix} \sum\nu_{j }\Delta_{f} G^{\circ}\text{(Reactants)=}4\times\Delta_{f} G^{o}_{Al^{3+}(aq)}+6\times\Delta_{f} G^{o}_{F^{-}(aq)}+12\times\Delta_{f} G^{o}_{OH^{-}(aq)} \\ =4\times489.4\text{+6}\times278.8\text{+12}\times157.24=-5517.28kJ\cdot mol^{-1} \end{matrix}$$

$$\Delta_{r} G^{\circ}=-5971.0+5517.28=-435.72kJ\cdot mol^{-1}<0$$

The calculated $\Delta_{r} G^{\circ}=-435.72kJ\cdot mol^{-1}$, which is far less than 0, indicating that the reaction is highly spontaneous under standard conditions (298.15 K, 1 bar).
